# Accelerated Radiotherapy Alone Versus Chemoradiation in Locally Advanced Carcinoma Cervix: Long-Term Outcomes

**DOI:** 10.7759/cureus.65154

**Published:** 2024-07-22

**Authors:** Vandana Singh Kushwaha, Kirti Srivastava, Sunil Kumar, Sandip Kumar Barik

**Affiliations:** 1 Radiation Oncology, All India Institute of Medical Sciences, Nagpur, Nagpur, IND; 2 Radiotherapy, King George's Medical University, Lucknow, IND; 3 Radiodiagnosis, King George's Medical University, Lucknow, IND; 4 Radiation Oncology, All India Institute of Medical Sciences, Bhubaneswar, Bhubaneswar, IND

**Keywords:** gyne-oncology, overall treatment time (ott), six fraction per week radiotherapy, predictors of survival, overall survival (os), acute toxicity, cancer cervix, accelerated radiotherapy, locally advanced carcinoma cervix, concurrent chemo-radiation

## Abstract

Introduction

Chemoradiation (CRT) is the standard of care for the treatment of carcinoma cervix, more benefits of CRT are seen in the early stage as compared to a locally advanced stage. Altered fractionation such as accelerated radiotherapy (ART) in locally advanced carcinoma cervix has not been explored much. Here, we have reported the long-term outcome of ART in comparison to conventional CRT in locally advanced cervical cancer patients.

Methods

From September 2011 to January 2014, 191 patients with locally advanced squamous cell carcinoma of the uterine cervix, FIGO stage IIB - IIIB were included in this study. They were randomized into two arms: the CRT arm (95 patients) versus the ART arm (96 patients). During external beam radiotherapy (EBRT), the patients in the CRT arm received conventional radiotherapy 50 Gy/25 fractions, 2 Gy/fraction, 5 fractions/week with cisplatin 40 mg/m^2^/week while patients in the ART arm received 50 Gy/25 fractions, 2 Gy/fraction, 6 fractions per week (Monday to Saturday) radiation alone. This was followed by three insertions of 6.5 Gy per fraction of high dose rate (HDR) brachytherapy at one-week intervals in both arms to keep the total treatment time 50 days in the CRT arm versus 45 days in the ART arm.

Results

The median follow-up of the study population was 57 months (range: 4-108 months). The patients with no residual disease (NRD) after EBRT and complete response (CR) at first follow-up were statistically less in the ART arm as compared to the CRT arm (30.2% versus 53.7% and 42.7% versus 63.2%; p = 0.006 and p = 0.024, respectively). However, there was no statistical difference in response at six months. High-grade acute toxicities hematological (9.5%) and gastrointestinal (15.8%) were more prevalent in the CRT arm in comparison to the ART arm, with no statistical significance (p>0.05) and Grade 1/2 genitourinary toxicity was significantly higher in the CRT arm. Late toxicities in both groups were equivalent. Recurrence, distant type of recurrence, and time to recurrence were similar in both groups. Five-year rates of overall survival (OS) and disease-free survival (DFS) were 51.2% versus 37.2% (p = 0.087) and 57.1% versus 46.3% (p = 0.223) in the CRT arm versus ART arm, respectively.

Conclusion

ART is a compelling alternative to concurrent chemoradiotherapy for locally advanced cervical cancer, particularly in patients with significant comorbidities, elderly women, and those in higher stages where concurrent chemotherapy's efficacy diminishes. It should be strongly considered when chemotherapy is contraindicated.

## Introduction

In India, approximately 1,24,000 new cases of cervical cancer are diagnosed with 77,000 cancer-related deaths every year [1]. Over 60% of patients are diagnosed with locally advanced stage according to the Report of the National Cancer Registry Programme (ICMR-NCDIR), India 2020 [2]. Cervical cancer is a leading cause of death and illness among women in India and low to middle-income countries. This is due to advanced-stage diagnosis, poor overall health, and low socioeconomic status, making it difficult for patients to afford even basic radiotherapy expenses [3].

Numerous studies have been conducted to improve locoregional control and survival in carcinoma cervix patients by various strategies such as adding chemotherapy, hyperbaric oxygen, hyperthermia, etc. Among them, chemoradiotherapy (CRT) has shown significant benefits in both survival and disease control years with increased high-grade toxicities over radiotherapy alone.

Chemo‐radiotherapy is perhaps the standard of care in the treatment of cervical cancer. Five randomized controlled trials have demonstrated a survival benefit for concurrent chemoradiation (CRT) over radiotherapy alone. This led to the 1999 National Cancer Institute (NCI) alert, which strongly advised that concurrent chemotherapy (cisplatin-based) should be considered for all patients with cervical cancer requiring radiotherapy [4]. It is worth remembering that these results were obtained in a trial setting, in women from affluent countries. The study population of the five trials was heterogeneous in stage, chemotherapy dosing, and scheduling with different radiation fields, and none of them were exclusively sketched for locally advanced stage (NCI trials) [5]. The effectiveness of CRT for locally advanced cases is still challenging. A Cochrane meta-analysis comparing CRT to radiation alone highlighted the benefits of adding chemotherapy. It showed benefits across various patient groups based on age, histology, grade, and pelvic node involvement. However, the survival advantage decreases with higher tumor stages, for instance: the five-year survival rates drop from 10% for stages IB-IIA to 7% for stage IIB and further decrease to 3% for stages III-IVA. With this trend, applying the overall hazard ratio of 0.81 to each stage subgroup suggests improved five-year survival across all stages, confirming the benefit of chemoradiotherapy for women with all stages of cervical cancer, though the degree of benefit varies by stage [6]. Hence, it is essential to thoroughly assess the benefits of adding concurrent chemotherapy to radiotherapy, considering safety, cost-effectiveness, and resource limitations. Exploring alternative radiotherapy modalities or variations is crucial for improving treatment efficacy, particularly in advanced cervical carcinoma cases. The standard treatment protocol involves external beam radiotherapy (EBRT) with concurrent chemoradiotherapy (CRT), followed by a brachytherapy boost to the cervix. Overall treatment time (OTT) is defined as the time duration from the first fraction of EBRT to the date of completion of ICRT. Several studies have described lower pelvic tumor control and survival rates in invasive uterine cervix carcinoma when the OTT is prolonged [7-13]. ART is a regimen intended to shorten the OTT while keeping the same total radiation dose and dose per fraction. The goal is to minimize tumor cell repopulation during treatment and to enhance the therapeutic ratio.

Six fractions per week of radiotherapy is the most attractive because it theoretically incurs a lower risk of excessive late toxicity, less demand for extra resources, and quicker turnover of patients. It also cuts short the overall stay of the patient and thereby increases treatment compliance. It is already been assessed in a phase II trial by Yoon et al. (2006), which demonstrated that six fractions per week of EBRT is an effective treatment option for patients with a carcinoma of the uterine cervix [14].

Our study aimed to achieve similar survival benefits by reducing OTT through ART, in comparison to concurrent CRT. Its purpose is to compare survival and toxicity outcomes between ART alone and concurrent CRT in patients with locally advanced cervical carcinoma.

## Materials and methods

**Figure 1:**
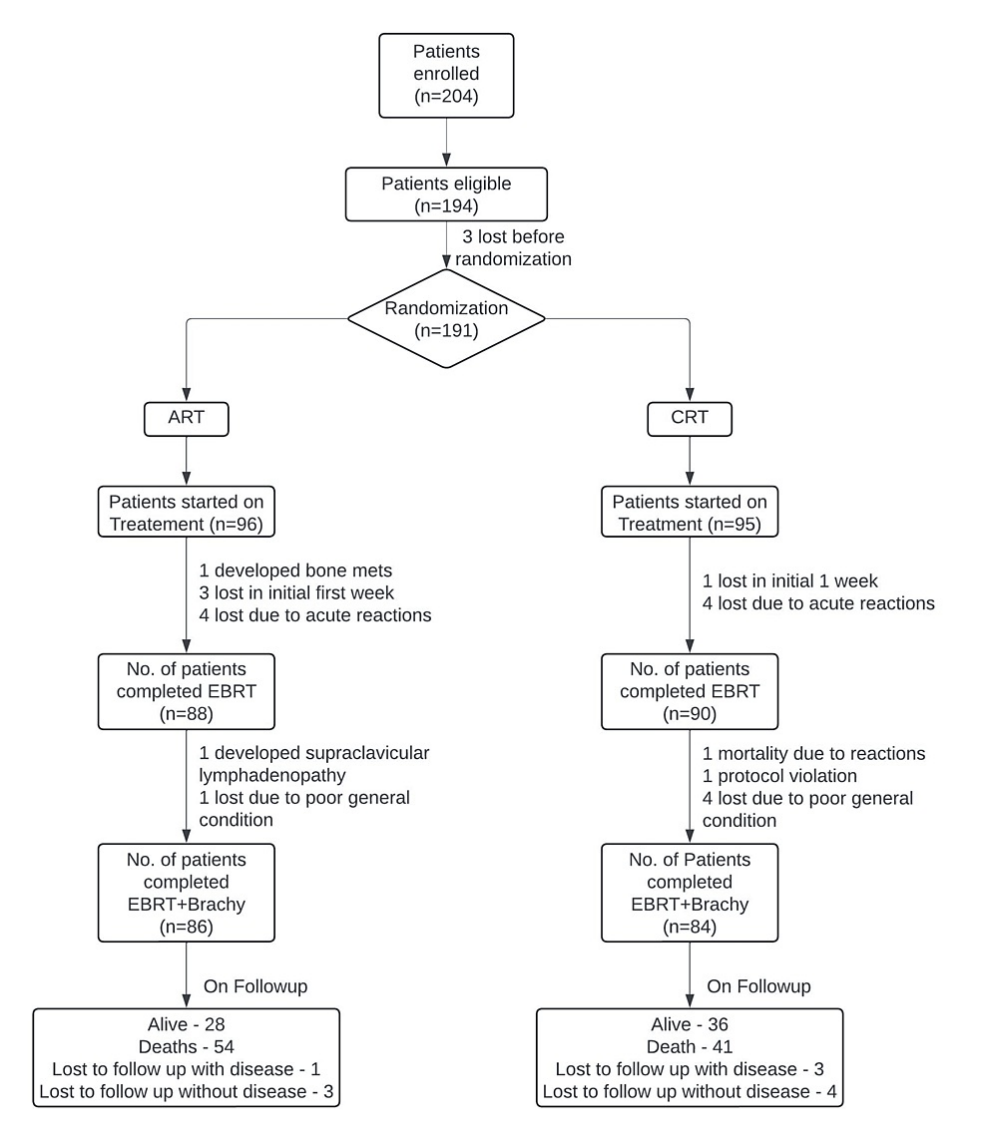
Consort diagram of the study showing the two treatment arms arm A: accelerated radiotherapy (ART); arm B: concurrent chemoradiation (CRT)

From September 2011 to January 2014, we conducted a prospective randomized study at the Department of Radiotherapy, King George’s Medical University (KGMU), Lucknow, India, enrolling 204 patients with histologically proven invasive squamous cell carcinoma of the cervix. Eligible patients, aged 70 or younger, with stages IIB-IIIB FIGO 2009, normal hematological, renal, and hepatic function, Karnofsky performance status ≥70, and no prior chemotherapy, radiotherapy, or surgery, were enrolled. The Institutional Ethics Committee of KGMU approved the study, and all patients provided written informed consent. Physical and pelvic examinations, baseline investigations, and radiological assessments (chest X-ray, abdominal CT scan, or ultrasound) were conducted. Cystoscopy was performed in cases with suspected bladder involvement. Out of 204 patients, 191 were eligible and randomized into two arms (Figure 1).

Control arm: CRT

Patients were given EBRT 50 Gy in 25 fractions, 200 cGy per fraction, 5 fractions per week with concurrent cisplatin 40 mg/m^2 ^(maximum dose up to 50 mg) per week followed by three insertions of intra-cavitary high dose rate (HDR) brachytherapy 6.5 Gy/fraction one week apart.

Study arm: ART

**Figure 2:**
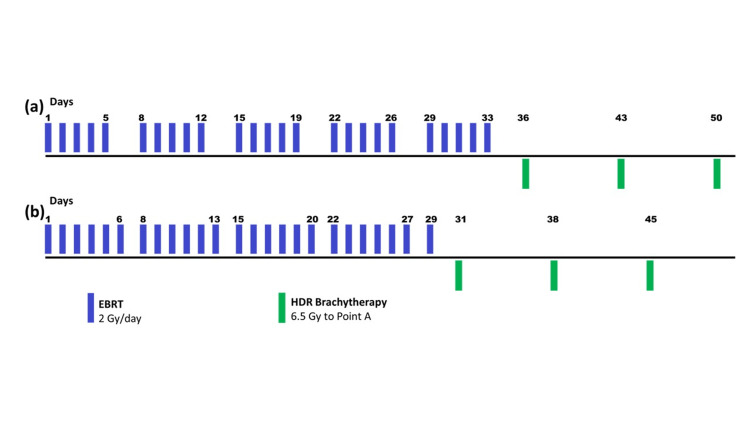
Treatment schema for patients with carcinoma cervix of both the arms in present study. (a) Conventional five fractions per week radiotherapy; (b) Experimental six fractions per week accelerated radiotherapy followed by weekly high dose rate (HDR) brachytherapy EBRT: external beam radiotherapy

Patients were given EBRT 50 Gy in 25 fractions, 200 cGy per fraction, 6 fractions per week followed by three insertions of intra-cavitary HDR brachytherapy 6.5 Gy/fraction one week apart (Figure 2).

Treatment details

All patients received EBRT by Co60 machine 50 Gy in 25 fractions, 200 cGy per fraction by anteroposterior-posteroanterior technique (AP-PA) or four-field box techniques to the whole pelvis. EBRT was followed by three insertions of intracavitary high-dose-rate radiotherapy (ICRT) 6.5Gy/fraction to point A. ICRT started just after the completion of EBRT in both arms. Three such fractions were given at one-week intervals using Fletcher-Suit (tandem and ovoids) after loading applicators. Orthogonal films were taken to verify the placement of applicators and to perform the dosimetric plan. All patients received a cumulative dose of 78 Gy equivalent doses in 2 Gy (EQD2) to point A using a microSelectron remote afterloader with an Iridium 192 radioactive source.

Patients were assessed every week during treatment for toxicities. Weekly hemograms and complete blood biochemistry for renal function were done in all patients. The Radiation Therapy Oncology Group (RTOG) criteria was used for radiation-induced toxicities. Response assessment was done at six weeks on completion of radiotherapy and a second overall response assessment was done at six months. Response assessment was done by both pelvic examination and CT scan abdomen-pelvis and was compared with diagnostic scan and pre-treatment findings. Treatment response was assessed according to Response Evaluation Criteria in Solid Tumors (RECIST) version 1.1 and divided into complete, partial, stable, and progressive diseases. Patients were also assessed clinically at the first insertion of ICRT after completion of EBRT for disease status and categorized as Gross residual disease if the tumor had not responded or progressed, Residual disease if partial response was there, and no residual disease (NRD) if there was complete response (CR).

All patients were followed up every month for the initial three months followed by every three months for two years, every six months for the next three years, and yearly thereafter. Regular follow-up was done to assess the disease status (disease-free status, residual, metastasis, and recurrence). Telephonic and postal follow‑up was also done to ascertain the recent status of the disease or date of death wherever applicable.

Biological equivalent dose (BED)

We attempted to calculate the biologically equivalent dose received by the tumor by EBRT in both treatment arms. We have used Fowler’s formula to calculate the BED which takes into account the continuous tumor repopulation, total treatment duration (T), and the potential doubling time (Tpot) [15,16]. The BED is given by the following formula:

$\text{BED} = nd \left[ 1 + \frac{d}{\alpha/\beta} \right] - \left( \frac{\ln 2}{\alpha \cdot T_{\text{pot}}} \right) (OT - T_k)$

where n is the number of fractions; d is the dose per fraction; α and β are the parameters of the LQ model. α/β is a measure of how a specific tissue will respond to fractionation and dose rate; OT is the total duration of treatment and T_k_ is the time from when repopulation starts. It is called the time of delayed repopulation; T_pot_ is the potential doubling time of clonogenic cells; ln 2 = 0.693 and constant values representative of rapidly proliferating tumors like cervical cancer are α/β = 10; α = 0.3 Gy-1; T_k_= 21 days; T_pot_ = 5 days [16].

Fowler found an equivalent chemotherapy-adjusted BED of 7.2 Gy in head and neck cancers, representing a 10% advantage over the total radiation dose [17]. Extrapolating this, a 5 Gy advantage was considered for the CRT arm. Calculating BED using the Fowler formula, the ART arm totaled 56.8 Gy over 28 days, while the CRT arm BED was 54.5 Gy over 33 days. Accounting for treatment times with the additional 5 Gy from chemotherapy, the final reached BED in the CRT arm was 59.5 Gy, highlighting the incremental benefit of concurrent chemotherapy in optimizing radiation therapy outcomes. However, this difference of 2.7Gy is a minute and comparable.

Statistical analysis

Continuous data were summarized in mean ± standard deviation (SD) whereas discreet (categorical) in number (n) and percentage (%). Continuous two independent groups (CRT arm and ART arm) were compared by independent Student’s t-test where categorical by chi-square (χ^2^) test. The five-year survival and disease-free survival between two independent groups were analyzed using the Kaplan-Meier method and compared by a log-rank test. Univariate (unadjusted) and multivariate (adjusted) time and event-dependent Cox proportional hazard regression was done to assess independent predictors of poor survival. A two-tailed (α = 2) p < 0.05 was considered statistically significant. All statistical analyses were performed using IBM SPSS Statistics for Windows, Version 20 (Released 2011; IBM Corp., Armonk, New York, United States).

## Results

Baseline characteristics

The baseline characteristics of the two groups (CRT arm and ART arm) are summarized in Table 1.

**Table 1:** Baseline characteristics of two groups. The age, KPS, EBRT duration, OTT, and total chemo cycles of the two groups were summarized in mean ± SD and compared by Student’s t-test, whereas stage, HPE, EBRT completed, and ICRT completed were summarized in number (n) and percentage (%) and compared by χ2 test. The number mentioned in parenthesis in the subscript represents the number of subjects KPS: Karnofsky’s performance score; HPE: histopathological examination; MD: moderately differentiated; PD: poorly differentiated; WD: well-differentiated; EBRT: external beam radiotherapy; ICRT: intracavitary radiotherapy; OTT: overall treatment time; CRT: chemoradiation; ART: accelerated radiotherapy

Baseline characteristics	Control arm CRT (n = 95) (%)	Study arm ART (n = 96) (%)	t/χ^2^ value	p-value
Age (yrs)	50.24 ± 9.63	49.88 ± 10.24	0.26	0.799
KPS (score)	84.95 ± 5.03	84.69 ± 5.02	0.36	0.721
Stage				
IIB	(45.3) 43	(49.0) 47	0.36	0.836
IIIA	(4.2) 4	(3.1) 3		
IIIB	(50.5) 48	(47.9) 46		
HPE				
MD	(54.7) 52	(52.1) 50	6.17	0.103
PD	(6.3) 6	(4.2) 4		
WD	(22.1) 21	(35.4) 34		
Unknown	(16.8) 16	(8.3) 8		
EBRT completed				
Yes	(94.7) 90	(91.7) 88	0.71	0.4
Defaulted	(5.3) 5	(8.3) 8		
ICRT completed				
Yes	(88.4) 84	(89.6) 86	0.07	0.797
Defaulted	(11.6) 11	(10.4) 10		
EBRT duration (days)	36.98 ± 3.67 (90)	32.05 ± (88) 3.08	9.71	<0.001
OTT (days)	58.18 ± 6.39 (84)	51.50 ± (86) 5.52	7.3	<0.001
Total chemotherapy cycles (no)	3.73 ± 1.03	-	NA	-

The two groups were matched (p > 0.05) with age, KPS score, stage, and HPE. Further, 90 (94.7%) patients in the CRT arm and 88 (91.7%) patients in the ART arm completed the EBRT. Similarly, 84 (88.4%) patients in the CRT arm and 86 (89.6%) patients in the ART arm completed ICRT. The frequency (%) of EBRT and ICRT completed was also found similar (p > 0.05) between the two groups. The mean EBRT duration and OTT were 36.98 ± 3.67 days versus 32.05 ± 3.08 days and 58.18 ± 6.39 days versus 51.50 ± 5.52 days in the CRT arm and the ART arm, respectively (p < 0.001).

Treatment response

The treatment response of the two groups is summarized in Table 2.

**Table 2:** Treatment responses of two groups. The time to achieve CR of the two groups was summarized as mean ± SD and compared by Student’s t-test, whereas disease status after EBRT, response at the first follow-up, and response at the six-month stage were summarized in number (n) and percentage (%) and compared by χ2 test. The number mentioned in parenthesis in the subscript represents the number of subjects GRD: gross residual disease; NRD: no residual disease; RD: residual disease; CR: complete response; PD: progressive disease; PR: partial response; SD: stable disease; CRT: chemoradiation; ART: accelerated radiotherapy

Treatment response	Control arm CRT (n = 95) (%)	Study arm ART (n = 96) (%)	t/χ^2^ value	p-value
Disease status (after EBRT)				
GRD	8 (8.4)	13 (13.5)	12.52	0.006
NRD	51 (53.7)	29 (30.2)		
RD	25 (26.3)	44 (45.8)		
Defaulted	11 (11.6)	10 (10.4)		
Response (at first follow-up)				
CR	60 (63.2)	41 (42.7)	11.27	0.024
PD	1 (1.1)	4 (4.2)		
PR	21 (22.1)	34 (35.4)		
SD	2 (2.1)	7 (7.3)		
Defaulted	11 (11.6)	10 (10.4)		
Response (at six months)				
CR	70 (73.7)	67 (69.8)	0.87	0.649
PD	14 (14.7)	19 (19.8)		
Defaulted	11 (11.6)	10 (10.4)		
Time to achieve CR (month)	1.71 ± 0.53 (70)	2.31 ± 1.26 (67)	3.61	<0.001

The frequency (%) of patients with NRD status after EBRT was significantly lesser (23.5%; p = 0.006) in the ART arm (30.2%) when compared to the CRT arm (53.7%). Further, the CR at the first follow-up also lowered (20.5%) significantly (p = 0.024) in the ART arm as compared to the CRT arm. Moreover, the mean time to achieve complete CR at six months was found significantly (p < 0.001) different and higher (25.7%) in the ART arm as compared to the CRT arm. However, responses at six months were found similar (p > 0.05) between the two groups i.e., did not differ significantly.

Toxicity

Acute Toxicity

The treatment-related acute toxicity of the two groups is summarized in Table 3.

**Table 3:** Treatment-related acute toxicity observed in the two treatment groups. The acute toxicity of the two groups were summarized in numbers (n) and percentages (%) and compared by the χ2 test CRT: chemoradiation; ART: accelerated radiotherapy

Acute toxicity	Control arm CRT (n = 95) (%)	Study arm ART (n = 96) (%)	χ^2^ value	p-value
Hematological				
Grade 0	50 (52.6)	61 (63.5)	4.53	0.21
Grade 1	20 (21.1)	16 (16.7)		
Grade 2	16 (16.8)	16 (16.7)		
Grade 3	9 (9.5)	3 (3.1)		
Gastrointestinal				
Grade 0	29 (30.5)	26 (27.1)	6.56	0.161
Grade 1	24 (25.3)	25 (26.0)		
Grade 2	27 (28.4)	39 (40.6)		
Grade 3	14 (14.7)	6 (6.3)		
Grade 5	1 (1.1)	0 (0)		
Genitourinary				
Grade 0	72 (75.8)	86 (89.6)	6.43	0.04
Grade 1	15 (15.8)	6 (6.3)		
Grade 2	8 (8.4)	4 (4.2)		
Skin				
Grade 0	86 (90.5)	85 (88.5)	0.2	0.654
Grade 1	9 (9.5)	11 (11.5)		

In comparison, the hematological, gastrointestinal, and skin toxicity were found similar (p > 0.05) between the two groups. However, genitourinary toxicity (grade 1 + grade 2) lowered (13.8%) significantly (p = 0.040) in the ART arm as compared to the CRT arm.

Late Toxicity

The treatment-related late toxicity of the two groups is summarized in Table 4.

**Table 4:** Treatment-related late toxicity observed in the two treatment groups. The late toxicity of the two groups were summarized in numbers (n) and percentages (%) and compared by the χ2 test CRT: chemoradiation; ART: accelerated radiotherapy

Late toxicity	Control arm CRT (n = 95) (%)	Study arm ART (n = 96) (%)	χ^2^ value	p-value
Rectum				
Grade 0	63 (66.3)	64 (66.7)	0.36	0.986
Grade 1	10 (10.5)	11 (11.5)		
Grade 2	8 (8.4)	9 (9.4)		
Grade 3	3 (3.2)	2 (2.1)		
Defaulted	11 (11.6)	10 (10.4)		
Bladder				
Grade 0	73 (76.8)	73 (76.0)	0.33	0.988
Grade 1	5 (5.3)	6 (6.3)		
Grade 2	4 (4.2)	4 (4.2)		
Grade 3	2 (2.1)	3 (3.1)		
Defaulted	11 (11.6)	10 (10.4)		
Small Bowel				
Grade 0	71 (74.7)	71 (74)	3.79	0.436
Grade 1	6 (6.3)	7 (7.3)		
Grade 2	6 (6.3)	3 (3.1)		
Grade 3	1 (1.1)	5 (5.2)		
Defaulted	11 (11.6)	10 (10.4)		
Vaginal				
Grade 0	66 (69.5)	64 (66.7)	0.53	0.971
Grade 1	13 (13.7)	16 (16.7)		
Grade 2	3 (3.2)	4 (4.2)		
Grade 3	2 (2.1)	2 (2.1)		
Defaulted	11 (11.6)	10 (10.4)		

On comparing, the late toxicity viz. rectum, bladder, small bowel, and vaginal were found similar (p > 0.05) between the two groups i.e., did not differ significantly.

Recurrence

The recurrence, type of recurrence, site of recurrence, and time to local recurrence (i.e., distant metastasis) of the two groups are summarized in Table 5.

**Table 5:** Distribution and patterns of recurrence observed in the two treatment groups. The time to local recurrence/distant Mets of two groups were summarized in mean ± SD and compared by Student’s t-test, whereas type of recurrence and site of recurrence were summarized in number (n) and percentage (%) and compared by χ2 test The number mentioned in parenthesis represents the number of subjects. CRT: chemoradiation; ART: accelerated radiotherapy

Recurrence	Control arm CRT (n = 95) (%)	Study arm ART (n = 96) (%)	t/χ^2^ value	p-value
Recurrence				
Recurrence-free	41 (43.2)	30 (31.3)	3.47	0.324
Never disease free	14 (14.7)	19 (19.8)		
Recurred	29 (30.5)	37 (38.5)		
Defaulted	11 (11.6)	10 (10.4)		
Type of recurrence				
Distant	10 (10.5)	19 (19.8)	3.99	0.263
Local	16 (16.8)	13 (13.5)		
Local and distant	3 (3.2)	5 (5.2)		
Defaulted and not recurred	66 (69.5)	59 (61.5)		
Site of recurrence				
Bone	1 (1.1)	3 (3.1)	8.11	0.323
Liver	0 (0.0)	1 (1.0)		
Local	16 (16.8)	13 (13.5)		
Local and distant	3 (3.2)	5 (5.2)		
Lung	2 (2.1)	1 (1.0)		
Multiple sites	6 (6.3)	7 (7.3)		
Para-aortic	1 (1.1)	7 (7.3)		
Defaulted	66 (69.5)	59 (61.5)		
Time to local recurrence (month)	23.17 ± 22.55 (29)	22.89 ± 21.30 (37)	0.05	0.959

The control arm (n = 95) showed 43.2% recurrence-free, 14.7% never disease-free, 30.5% recurrence, and 11.6% defaulted, while the study arm (n = 96) exhibited 31.3% recurrence-free, 19.8% never disease-free, 38.5% recurrence, and 10.4% defaulted. Patterns of recurrence analysis revealed a 10.5% distant, 16.8% local, and 3.2% local and distant in the control arm, compared to 19.8% distant, 13.5% local, 5.2% local and distant in the study arm. Site of recurrence data included 16.8% local, 3.2% bone, 2.1% lung, 6.3% multiple sites, 1.1% para-aortic, and 69.5% defaulted in the control arm, and 13.5% local, 3.1% bone, 1% liver, 1% lung, 7.3% multiple sites, 7.3% para-aortic, and 61.5% defaulted in the study arm. On comparing, recurrence, type of recurrence, site of recurrence, and time to local recurrence were also found similar (p > 0.05) between the two groups i.e., did not differ significantly.

Patient status at last follow-up

The patients of the two groups were followed up for five years. The actual patient status and final status of patients were also found similar (p > 0.05) between the two groups (Table 6).

**Table 6:** Patient status on follow-up was observed in the two study groups. The patient’s status in two groups were summarized in numbers (n) and percentages (%) and compared by the χ2 test CRT: chemoradiation; ART: accelerated radiotherapy

Patient’s status	Control arm CRT (n = 95) (%)	Study arm ART (n = 96) (%)	t/χ^2^ value	p-value
Actual patient status				
Alive	36 (37.9)	28 (29.2)	4.62	0.328
Expired	41 (43.2)	54 (56.3)		
Lost to follow-up with disease	3 (3.2)	3 (3.1)		
Lost to follow-up without disease	4 (4.2)	1 (1.0)		
Defaulted	11 (11.6)	10 (10.4)		
Final status of patients				
Alive (censored)	43 (51.2)	32 (37.2)	3.37	0.066
Expired	41 (48.8)	54 (62.8)		

However, of a total of 84 patients in the CRT arm (after excluding 11 defaulters), 43 (51.2%) patients were alive or censored (alive + lost to follow up with disease + lost to follow up without disease) and 41 (48.8%) patients died due to disease. In contrast, of a total of 86 patients in the ART arm (after excluding 10 defaulters), 32 (37.2%) patients were alive or censored (alive + lost to follow up with disease + lost to follow up without disease), and 54 (62.8%) patients died due to disease. The ART arm, thus accounting for 14.0% more deaths as compared to the CRT arm, however, did not reach statistical significance.

Survival

**Figure 3:**
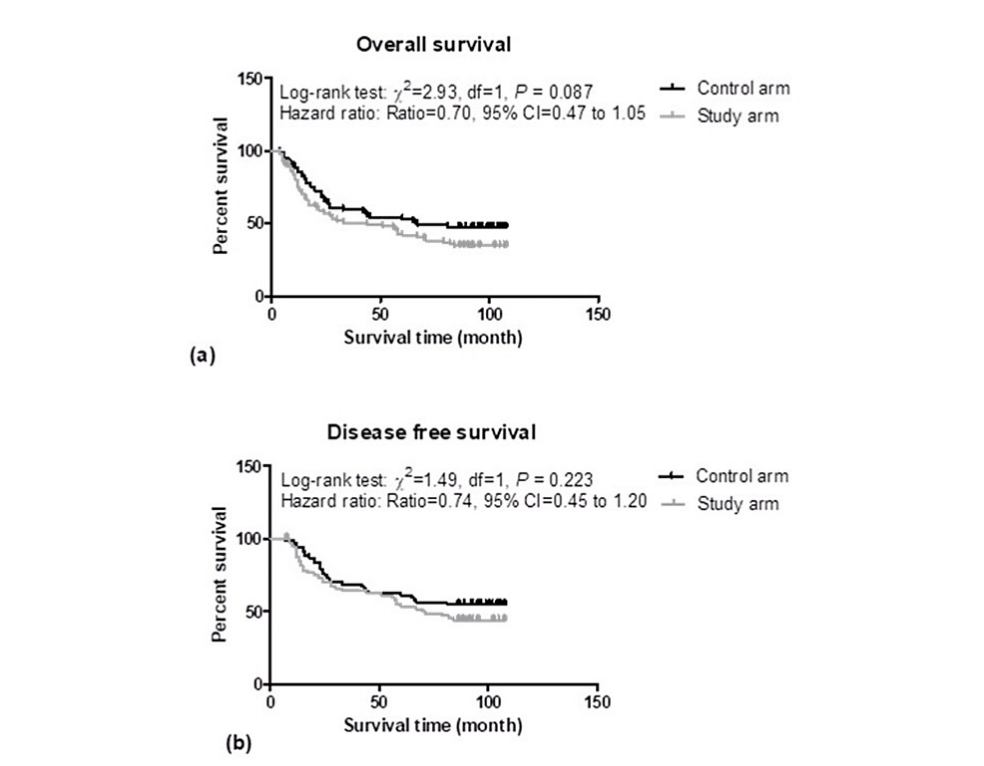
Kaplan Meir graphs showing the five years of overall survival (3A) and disease-free survival (3B) in both the study groups

The five-year overall disease-free and recurrence-free survivals of the two groups are summarized graphically in Figure 3.

In five years of overall survival, there were 41 (48.8%) patients died due to disease and 43 (51.2%) alive in the CRT arm with a median survival of 66 months whereas in the ART arm, 54 (62.8%) died and 32 (37.2%) alive with median survival 44 months. Comparing the overall survivals of the two groups, the log-rank test showed similar overall survivals between the two groups (χ^2 ^=2.93, p = 0.087) though the death rate per month was 0.70 fold higher in the ART arm as compared to the CRT arm (hazard ratio = 0.70, 95% CI = 0.46 to 1.05) (Figure 3A).

Similarly, in five years of disease-free survival, there were 30 (42.9%) deaths and 40 (57.1%) alive in the CRT arm with a median survival of 86 months; whereas, in the ART arm, there were 36 (53.7%) deaths and 31 (46.3%) alive with median survival 67 months. Comparing the disease-free survivals of the two groups, the log-rank test showed similar disease survivals between the two groups (χ^2 ^= 1.49, p = 0.223) though the death rate per month was 0.74 fold higher in the ART arm as compared to the CRT arm (hazard ratio = 0.74, 95% CI = 0.45 to 1.20) (Figure 3B).

Predictors of poor survival

The predictors of survival among demographics (age and KPS score), findings (stage and HPE), treatment (EBRT, ICRT, EBRT duration, OTT), response (final disease status after EBRT, response at first- and sixth-month follow-up), toxicity (acute and late), and recurrence in all treated patients (CRT arm and ART arm) were evaluated using univariate (unadjusted) and multivariate (adjusted) Cox proportional hazard regression analysis and summarized in Table 7.

**Table 7:** Identification of predictors of poor survival (control arm + study arm, n = 170) using univariate and multivariate Cox proportional hazard regression analysis KPS: Karnofsky’s performance score; HPE: histopathological examination; MD: moderately differentiated; PD: poorly differentiated; WD: well-differentiated; EBRT: external beam radiotherapy; NRD: no residual disease; GRD: gross residual disease; RD: residual disease; CR: complete response; PR: partial response; SD: stable disease; PD: progressive disease; OTT: overall treatment time; OR: odds ratio; CI: confidence interval; Ref: reference group (the odds ratio was evaluated with respect to the reference group)

Predictors	Univariate (unadjusted)		Multivariate (adjusted)	
	OR (95% CI)	p-value	OR (95% CI)	p-value
Age (years)				
≤50	Ref			
>50	0.75 (0.50-1.13)	0.173		
KPS (score)				
≤80	Ref		Ref	
>80	0.59 (0.39-0.89)	0.012	1.14 (0.74-1.75)	0.555
Stage				
IIA	Ref			
IIIA + IIIB	1.40 (0.94-2.10)	0.099		
HPE				
Unknown + WD	Ref			
MD + PD	1.06 (0.70-1.58)	0.797		
EBRT duration (days)				
≤35	Ref			
>35	0.78 (0.51-1.21)	0.783		
OTT (days)				
≤55	Ref			
>55	1.10 (0.74-1.65)	0.64		
Final disease status (after EBRT)				
NRD	Ref		Ref	
GRD + RD	2.44 (1.60-3.72)	<0.001	1.34 (0.69-2.60)	0.391
Response at first follow-up				
CR	Ref		Ref	
PR + SD + PD	2.61 (1.74-3.91)	<0.001	0.87 (0.42-1.79)	0.697
Response at sixth-month follow-up				
CR	Ref		Ref	
PD	12.27 (7.22-20.86)	<0.001	4.55 (2.36-8.75)	<0.001
Hematological toxicity				
Grade 0	Ref			
Grade 1-3	1.39 (0.92-2.10)	0.115		
Gastrointestinal toxicity				
Grade 0	Ref			
Grade 1-5	1.02 (0.66-1.57)	0.943		
Genitourinary toxicity				
Grade 0	Ref			
Grade 1-2	1.65 (0.98-2.79)	0.061		
Skin toxicity				
Grade 0	Ref			
Grade 1	0.71 (0.33-1.53)	0.381		
Rectum toxicity				
Grade 0	Ref			
Grade 1-3	0.63 (0.39-1.02)	0.061		
Bladder toxicity				
Grade 0	Ref			
Grade 1-3	0.63 (0.35-1.16)	0.141		
Small bowel				
Grade 0	Ref			
Grade 1-3	0.63 (0.36-1.11)	0.109		
Vaginal toxicity				
Grade 0	Ref		Ref	
Grade 1-3	0.44 (0.26-0.75)	0.002	0.43 (0.24-0.76)	0.004
Recurrence				
Disease free	Ref		Ref	
Recurred + Never disease-free	41.43 (16.38-104.8)	<0.001	43.79 (16.70-114.81)	<0.001

The univariate analysis showed KPS, final disease status after EBRT, response at first- and sixth-month follow-up, vaginal toxicity, and recurrence were significant (p < 0.05, p < 0.01, or p < 0.001) risk predictors of poor survival. In multivariate analysis, the response at sixth-month follow-up, vaginal toxicity, and recurrence further showed significant (p < 0.01 or p < 0.001) association with poor survival and thus may be severed as significant and independent predictors of poor survival in carcinoma cervix-treated patients (CRT and accelerated radiation).

## Discussion

The established standard treatment for cervical cancer is radiation therapy with concurrent chemotherapy. For patients unable to undergo chemotherapy, radical radiation therapy alone is the default option. Despite standard approaches, local relapse remains a major treatment failure, highlighting the need to improve local therapy for better locoregional control. This study aims to compare the gold standard treatment regimen versus pure ART and provide an alternative approach for cervical cancer patients.

While concurrent chemoradiotherapy became the standard of treatment following the 1999 NCI clinical alert based on five randomized trials, a contemporary Canadian trial by Pearcey et al. focused exclusively on locally advanced stages found no benefit in adding chemotherapy to radical radiotherapy. Instead, it emphasized optimizing radiotherapy details and techniques to achieve the best treatment outcomes [18].

Other multicentric trials such as the study by Lee et al. have shown no benefit with concurrent chemoradiotherapy as compared to radiotherapy alone [19]. There are several recently conducted Indian studies that have failed to demonstrate a clear benefit of CRT over radiotherapy [20-22]. A study by Datta and Agrawal reported that although chemoradiotherapy could be a standard form of treatment for early cancer of the cervix, its role in advanced stages needs further exploration before this could be incorporated into routine clinical care, especially in developing countries [5]. The benefit of concurrent chemotherapy seemingly diminishes with increasing stage of the disease.

The addition of concurrent chemotherapy is also associated with a lot of toxicities. Acute hematological and gastrointestinal toxicity is significantly higher in patients receiving CRT in comparison to those receiving radiation alone. It has been hypothesized that the addition of chemotherapy to radiation as a radiosensitizer contributes to the observed significant improvement in survival and reduced recurrence rates. Several authors have attempted to quantify this effect of concurrent chemotherapy on the effect of radiation on tumor cells in terms of radiation dose and termed it the chemotherapy-adjusted BED [17,23]. Patients who are not fit to receive concurrent chemotherapy end up receiving radiation alone. Various attempts to compensate for this lack of chemotherapy-adjusted BED by varying the radiation dose fractionation schedules were explored in the recent past. Thus came the concept of an altered fractionation schedule for improving treatment outcomes with radiation alone. Various modifications in fractionation schedules have been tried like hyperfractionation and accelerated fractionation to enhance the locoregional CRT of the disease in locally advanced cases.

It has been observed that overall treatment beyond 56 days is associated with worse outcomes and a 17% increase in pelvic failures [24]. Additionally, studies by Maciejewski et al. and Withers et al. have shown that with increasing overall time the total dose to cure a tumor, had to be raised; this was attributed to tumor cell repopulation, which may not be important until the third week of a course of treatment [25,26]. Each extra day of treatment beyond a definite period, a dose increment of 0.6 Gy/day is required to overcome the accelerated repopulation [27]. Hence the authors' assumption while designing this study was that reducing OTT by giving six fractions per week (one extra fraction on Saturday) during EBRT would benefit from this dose requirement and combat the accelerated repopulation which ultimately results in better locoregional CRT and survival benefit. While considering the absolute dosimetric benefit of ART over standard five fraction per week radiotherapy not accounting for chemotherapy benefit was calculated using the simplified Fowler formula $\text{BED} = nd \left( 1 + \frac{d}{\frac{\alpha}{\beta}} \right) - 0.6 (T - 21)$ to around 2.3 Gy benefit of six fraction per week ART over five fraction per week radiotherapy alone [28]. However, our results show a significant difference in the OTT between the study (ART) arm 58.18 ± 6.39 days and the control (CRT) arm 51.50 ± 5.52 days (p < 0.001) which is approximately seven days. Now considering the benefit of 0.6 Gy/day, by reducing the OTT by seven days we were able to achieve a benefit of 4.2 Gy in the study (ART) arm over the control (CRT) arm which is equivalent to the 5 Gy benefit given by concurrent chemotherapy.

Our observed five-year overall survival was 37.2% in the ART arm and 51.2% in the CRT arm however not statistically significant. The five-year disease-free survival in the ART arm was comparable to that of the CRT arm, even though we didn’t find any difference in recurrence pattern and time to develop recurrence. This difference in survival rates can be attributed primarily to the shorter treatment time in the ART arm which was also observed in a previously conducted study by Kumar et al. [29]. Our results further bolster the observation that shortening the OTT in cancer cervix is associated with better outcomes.

Higher grade 1 and 2 toxicity was observed in the control arm (CRT) which was significantly higher than the study (ART) arm. The toxicity profile was found to be similar to that reported by Daripa et al. in their recent study [30]. This increased toxicity can be attributed to the addition of concurrent chemotherapy.

Until now, the exploration of altered fractionation in cervical carcinoma has been limited, with existing literature comprising only a few studies characterized by small sample sizes and brief follow-up periods. Our study stands out as one of the pioneering efforts, comparing ART to conventional fractionated radiotherapy in a significantly larger patient cohort, and providing insights into long-term outcomes and recurrence patterns over a five-year follow-up period.

Our study's constraints include the utilization of traditional AP-PA and four-field box radiotherapy methods instead of contemporary conformal treatment techniques, which might have reduced radiation toxicity even more.

## Conclusions

A new fractionation schedule was used in the present study, resulting in an appropriate shorter OTT. We did not find any statistically significant difference in survival outcome or recurrence between the two arms; the benefit of concurrent chemotherapy in the CRT arm was possibly balanced by the decrease in OTT in the ART arm. Patients tolerated both treatment regimens well, although higher-grade reactions were more common in the concurrent chemoradiotherapy (CRT) arm, as anticipated.

ART offers a highly promising option for treating locally advanced cervical cancer, especially in patients who face significant challenges with concurrent chemoradiotherapy. This approach can be considered for elderly women, individuals with substantial comorbidities, and patients for whom chemotherapy is ineligible. ART emerges as a compelling treatment strategy when chemotherapy presents contraindications due to medical reasons or patient-specific factors. Its role becomes pivotal in ensuring effective cancer management while navigating the complexities of individual health conditions and treatment tolerability.
